# The first use of laparoscopy to treat pelvic ring fractures: A case report

**DOI:** 10.1016/j.ijscr.2020.09.162

**Published:** 2020-09-25

**Authors:** Rémi Di Francia, Jean-Loup Tanner, Julien Marolleau

**Affiliations:** a Service de Traumatologie, CHRU de Brest, France; b Service d’Urologie, CHRU de Brest, France

**Keywords:** Laparoscopy, Pelvic ring fracture, Plate fixation

## Abstract

**Introduction:**

Pelvic ring injuries pose major challenges for the trauma surgeon. Given the complications of open surgeries, percutaneous techniques have become increasingly popular, but laparoscopy has never been employed for internal osteosynthesis of the pelvic ring.

**Presentation of case:**

We describe a 34-year-old male with a pelvic ring fracture (grade AO/OTA 61B2.1b). The anterior pelvic arch was successfully plate-fixed using a laparoscopic technique. This is the first report of such an approach to treat pelvic ring fractures.

**Discussion:**

Our principal point is that “laparoscopic internal fixation” delivered an *in situ* result as good as that of open surgery, but we required only four trocar portals.

**Conclusion:**

We describe a novel laparoscopic approach to treatment of pelvic ring fractures.

## Highlights

• The first use of laparoscopy to treat pelvic ring fractures: a case report.
• We describe the first use of laparoscopy to treat pelvic ring fracture.
• This “laparoscopic internal fixation” delivered an *in situ* result as good as that of open surgery.
• Despite a long operating time, the blood loss was very low.
• This technique opens a new approach to treatment of pelvic fractures.

## 1 Introduction

Pelvic ring injuries pose major challenges for the trauma surgeon [1]. Given the complications of open surgeries, percutaneous techniques have become increasingly popular [2]. However, such techniques cannot be used to fix the anterior arch of the pelvic ring. Laparoscopy is better than open surgery when treating digestive tract [3,4] and kidney conditions [5]. In the context of pelvic ring surgery, laparoscopy has been used only to extract material or to treat tissue migration [6]. To the best of our knowledge, laparoscopy has never been employed for internal osteosynthesis of the pelvic ring. We here describe the first use of laparoscopy to treat such a fracture.

## 2 Case and technique

This case report adheres to the SCARE checklist [7].

**Fig. 1:**
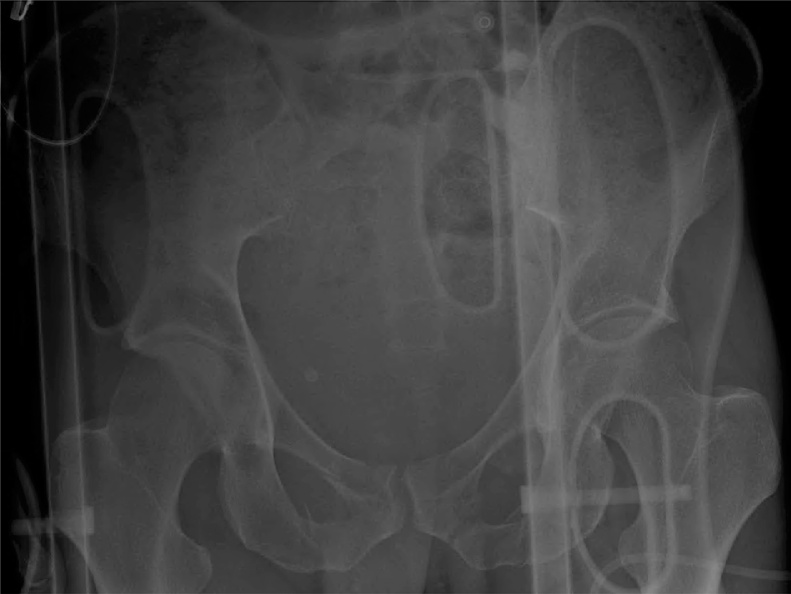
Pelvic anteroposterior X-rays taken before surgery.

**Fig. 2:**
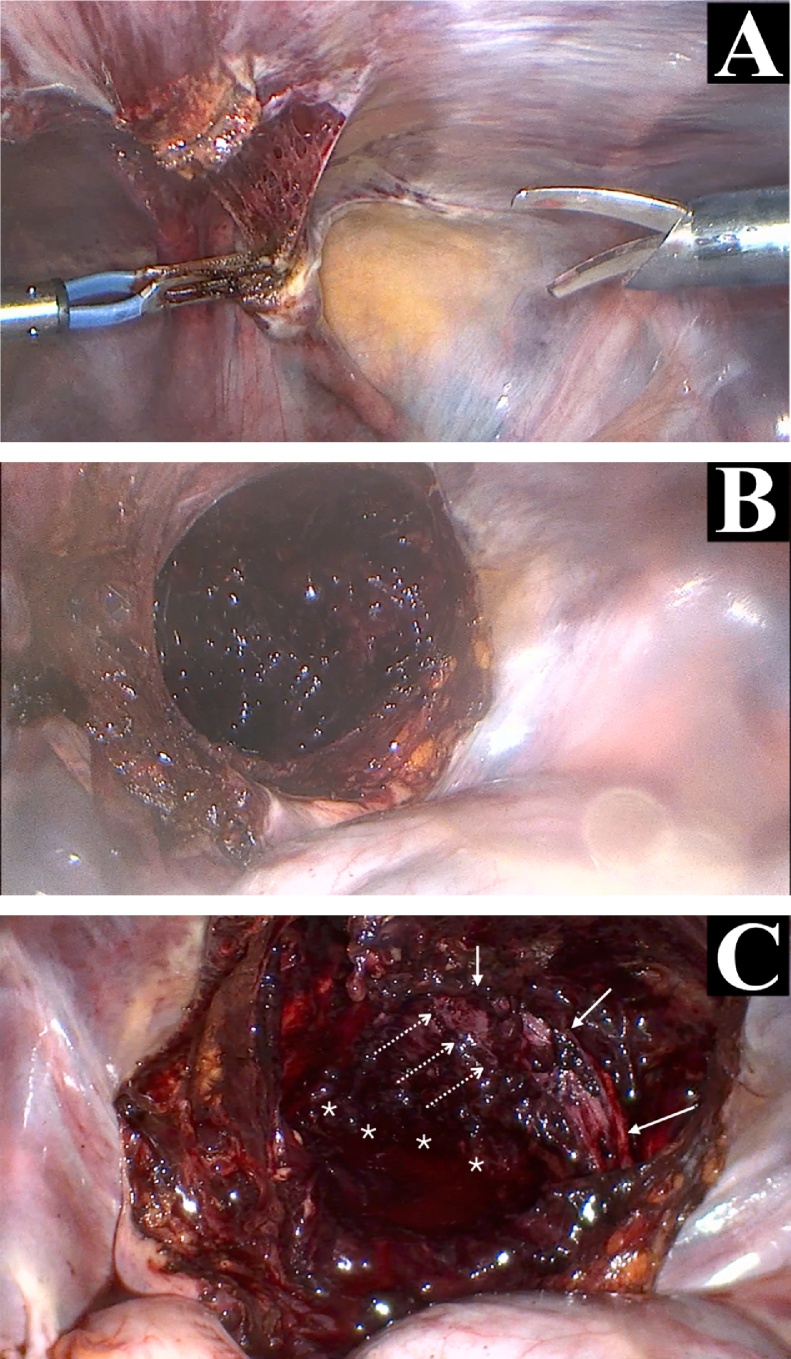
Laparoscopic views of the transperitoneal approach. Opening of the Retzius space using bipolar forceps and monopolar scissors (AB) allowing to reach pelvis (C), and see the right anterior arch (solid arrows), the fracture of the right obturator frame (hatched arrows), and the hematoma (*).

**Fig. 3:**
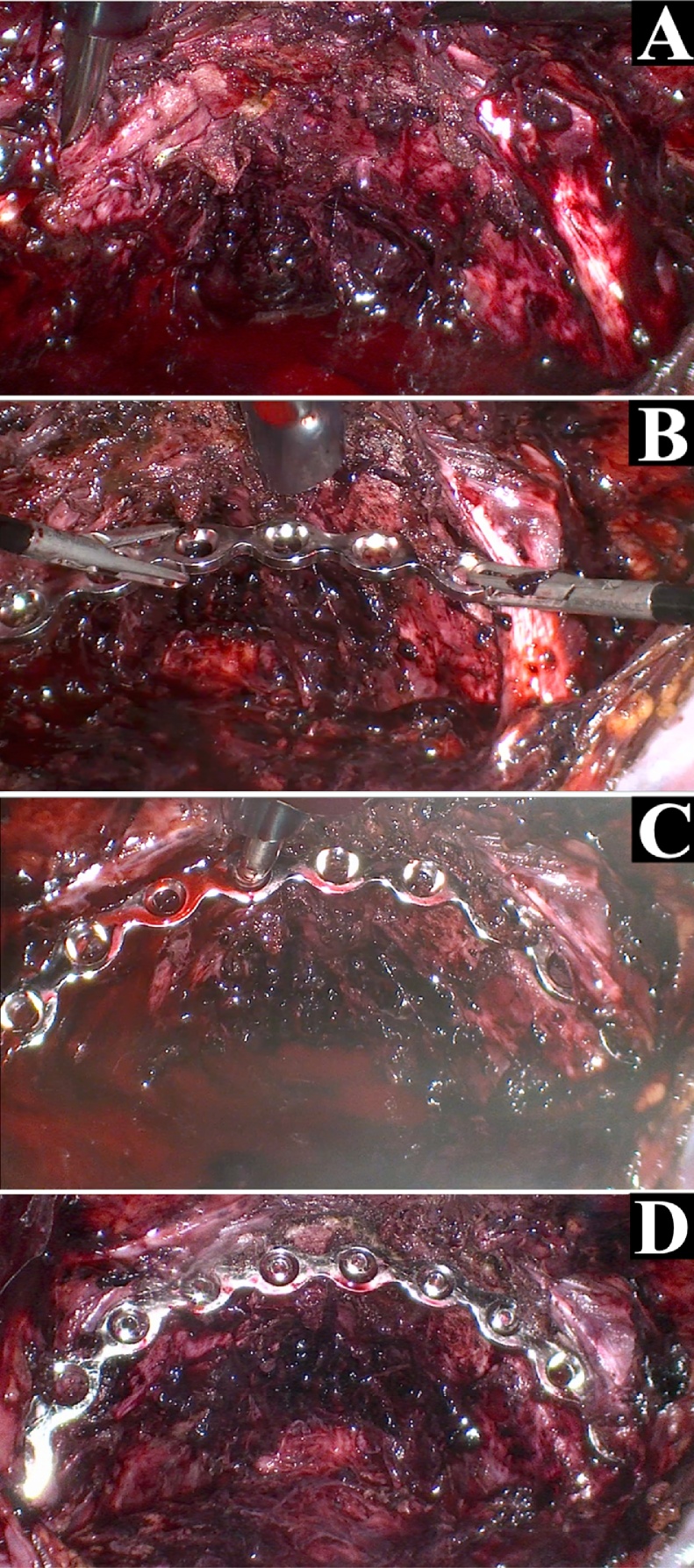
Laparoscopic views obtained after the pubic arch dissection (A), during the positioning of the plate using forceps (B) and the ball-spike pusher (C), and after screws insertion (D).

**Fig. 4:**
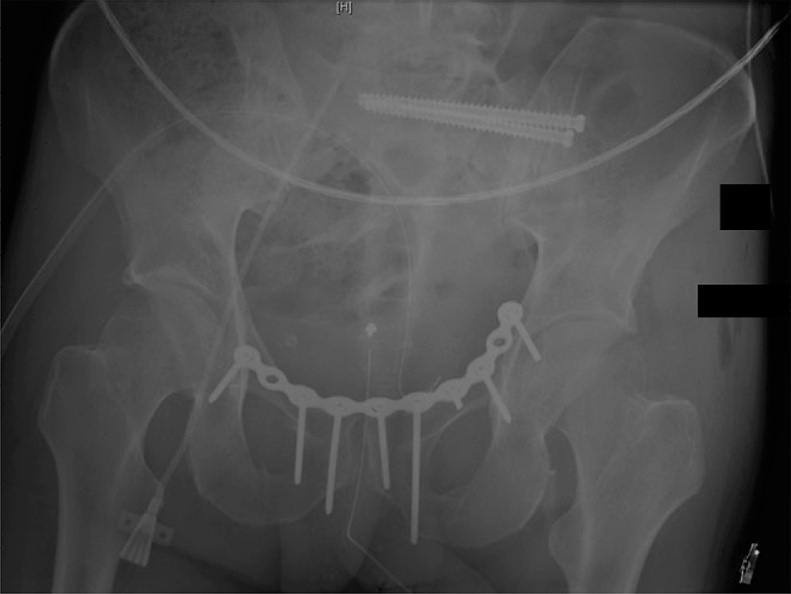
Pelvic anteroposterior X-rays taken after surgery.

A 34-year-old male without medical or surgical history was admitted to the intensive care unit of our level 1 trauma centre after a road accident. He had severe head and pelvic ring injuries. Emergency pelvic radiographs (Fig. 1) and computed tomography revealed a lateral compression fracture with internal rotational instability; fracture of the left sacral wing (Denis grade 1) was associated with bilateral fracture of the obturator bone frames (grade AO/OTA 61B2.1b). We decided to fix the posterior arch of the pelvic ring, but also the anterior arch, as recommended by the AO [8]. Surgery was performed by senior trauma (RDF) and urological (JM) surgeons. We first inserted two, left, sacroiliac cannulated screws (TIS; Königsee Implantate GmbH, Allendorf, Germany) via a classical percutaneous approach aided by inlet and outlet fluoroscopic views. We next performed laparoscopy. The peritoneal cavity was entered using an open laparoscopy technique via a 10/12-mm optical trocar positioned below the umbilicus. The pneumoperitoneum was insufflated to 12 mmHg and the intestinal loops repressed by placing the patient in the accentuated Trendelenburg position. Two further 10/12-mm trocars were added, triangulating the left and right pararectal areas. The Retzius space was opened using bipolar forceps and monopolar scissors (Fig. 2A). Dissection proceeded to the endopelvic fascia covering the base of the prostate (Fig. 2B-C). A fourth trocar (10/12 mm) was then created in the pubis to allow passage of instruments required for screw insertion. The pubic arch was carefully dissected. The right and left *coronae mortices* were ligated with bipolar forceps. Use of a monopolar hook and a dissector facilitated release of the pelvic bone surface. After dissection was adequate (Fig. 3A), a 10-hole, Matta curved plate (Stryker GmbH, Selzach, Switzerland) was inserted through a trocar and retrieved using forceps (Fig. 3B). When the plate was appropriately positioned on the anterior arch of the pelvic ring, it was fastened using a ball-spike pusher inserted into a trocar (Fig. 3C). A plate screw inserter (associated with a long drill guide) was then sequentially introduced into the various trocars to ensure appropriate orientation of all screws. The two key screws (those at the ends of the plate) were first inserted using a long screwdriver. When the plate was stably fixed to bone, the other screws were inserted. All screws required for reliable fixation were successfully inserted (Fig. 3D). Fluoroscopy was then performed. Given the space required by the laparoscopic instruments and surgical manipulation, screw placement under direct fluoroscopy was impossible. Surgery required 4 h; blood loss was less than 100 mL, reflecting, principally, evacuation of a hematoma. We encountered no intraoperative complications; the patient did not react negatively to laparoscopy; and the postoperative X-rays were satisfactory (Fig. 4). Unfortunately, the patient died from his head trauma 16 days after the accident.

## 3 Discussion

Our principal point is that “laparoscopic internal fixation” delivered an *in situ* result as good as that of open surgery, but we required only four trocar portals (Fig. 3). Management of pelvic ring fractures remains complex; the surgical approaches are risky. The Pfannenstiel and ilioinguinal approaches may injure the bladder, spermatic cord, iliac vessels, and the femoral and lateral thigh nerves; and/or trigger herniation [9]. Developments in percutaneous screws since the 1990s have reduced intraoperative comorbidities, blood loss, operative time, negative post-operative outcomes, decubitus complications, and the infection rate; and facilitate early mobilization [10,11]. However, percutaneous screwing remains somewhat risky and technically challenging [2,12]. Laparoscopy is better than open surgery when treating digestive tract and urological conditions [[3], [4], [5]], associated with low rates of intraoperative complications. Despite the longer operative time, blood loss and the hospital stay are reduced. In our case, blood loss was very low. The long operating time is explained by certain intrinsic difficulties; urological surgeons lack experience with laparoscopic dissection of the pelvic ramp. Plate handling and positioning are also challenging. Screw insertion is slower than that of open surgery because instrument manipulation within trocars is delicate. Trauma surgeons are often not familiar with spatial laparoscopic landmarks.

Our treatment requires the use of Stryker PRO Pelvis and Acetabulum System Instrumentation (Stryker GmbH, Selzach, Switzerland). Our success heralds a new approach to pelvic ring fractures; we will refine our technique further. The learning curve, especially that of operative time, requires attention.

## 4 Conclusion

We describe a novel laparoscopic approach to treatment of pelvic ring fractures.

## Declaration of Competing Interest

All authors declare that they have no conflict of interest.

## Funding

This research did not receive any specific grant from funding agencies in the public, commercial, or not-for-profit sectors.

## Ethical approval

Because of his death, the fact that the patient does not have to provide opposition in his lifetime, and in accordance with the French law "Informatique et libertés" of 2004 (Article 56), the use of health data of this patient in this case report does not require the approval of our local ethics committee.

## Consent

The patient was never able to give informed consent due to the sedation required since his accident, until his death.

We have provided a statement from our Head of Department that we have tried to contact the family on several times, and that the paper has been sufficiently anonymized not to cause harm to the patient or their family. (see below).

## Author’s contribution

J-L TANNER: data collection; literature review; writing.

R DI FRANCIA and J MAROLLEAU: operation.

R DI FRANCIA: translation, review, submission.

## Registration of research studies

researchregistry5934.

## Guarantor

Rémi DI FRANCIA.

Corresponding author.

## Provenance and peer review

Not commissioned, externally peer-reviewed.
